# Supplementary figures and images for: DZNep, an inhibitor of the histone methyltransferase EZH2, suppresses hepatic fibrosis through regulating miR-199a-5p/SOCS7 pathway
Source: PeerJ. 2021 May 14;9:e11374. doi: 10.7717/peerj.11374 (PMC8127960; doi:10.7717/peerj.11374)

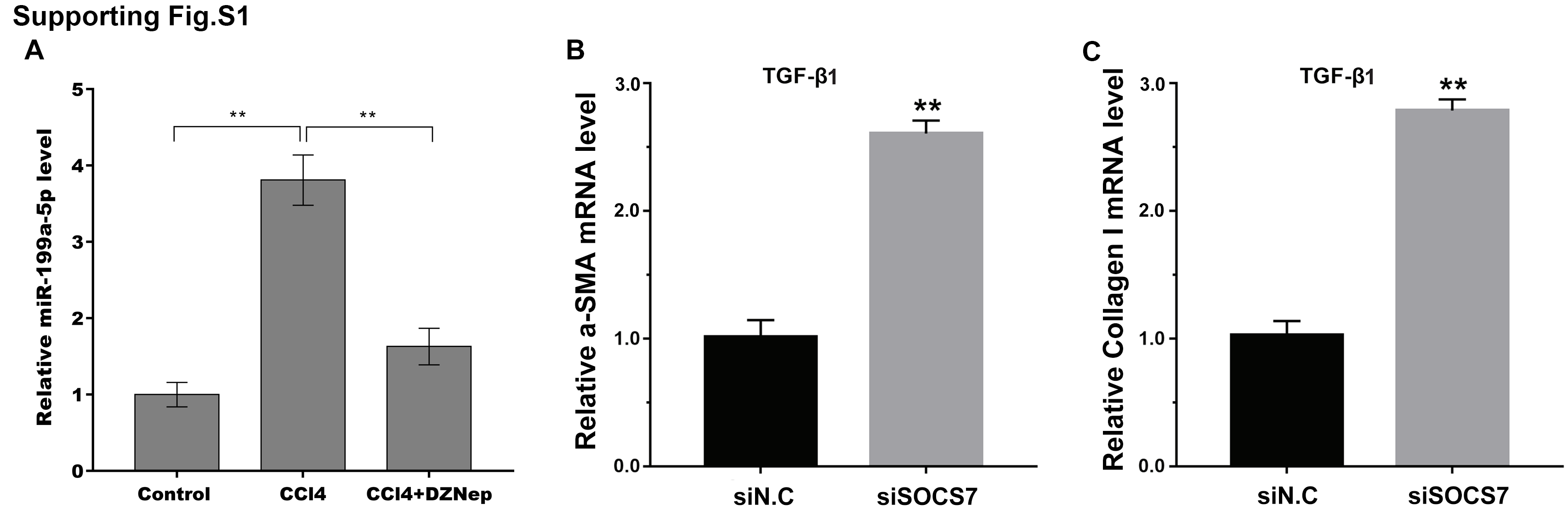

Supplement: Figure S1 [file peerj-09-11374-s003.png]
